# Translation and psychometric evaluation of the Chinese version of the Nursing Time Management Scale

**DOI:** 10.3389/fmed.2024.1396625

**Published:** 2024-05-10

**Authors:** Zhaoquan Fu, Yaping Wang, Limei Zhang, Mingyang Tan

**Affiliations:** ^1^Department of Health, Yantai Nanshan University, Yantai, China; ^2^Longkou Nanshan Health Valley Cancer Hospital, Yantai, China; ^3^Department of Nursing, Jinzhou Medical University, Jinzhou, China

**Keywords:** nursing, time management, reliability, validity, psychometric evaluation

## Abstract

**Background:**

Reasonable and effective time allocation can promote the improvement of medical care service quality. This study aimed to translate, cross-culturally adapt and validate the Chinese Nursing Time Management Scale (NTMS).

**Methods:**

Using a cross-sectional survey, 345 clinical nurses were selected from June to September 2023 for a general information questionnaire and Nursing Time Management Scale (NTMS) study. Item analysis, exploratory factor analysis and validation factor analysis were used to verify the reliability and validity of the Chinese version of Nursing Time Management Scale.

**Results:**

The Chinese version of the Nurses’ Time Management Competency Scale includes 17 entries in 3 dimensions: planning activities and setting goals, coordinating activities and procedures, and organizing nursing activities. The Cronbach’s alpha coefficient for the total scale was 0.966. Exploratory factor analysis showed that the cumulative variance contribution of the three male factors was 97.44%.

**Conclusion:**

The NTMS has acceptable validity and reliability and can be used to evaluate the nursing time management skills of Chinese clinical nurses.

## 1 Introduction

With the continuous progress and development of China’s economy and society, the level of people’s attention and concern for health is also increasing, and the compact and heavy nursing work requires nursing staff to accelerate the improvement of the efficiency of the use of time in order to meet the people’s needs for health (1). In today’s rapid development of science and technology, time management has become a science with high applicability and operability (2). Clinical nursing work is always faced with dealing with patients with acute, critical and severe diseases, as well as the need to deal with multiple patients at the same time, a number of complicated nursing tasks (3), so to become an excellent nurse not only need to master all the operational skills, but also need a kind of ability to rationally arrange time.

Time management is the process by which individuals, within a limited period of time, through the correct understanding of time and the rational arrangement of tasks, prompts people to change the use of time from a passive natural experience to a planned and purposeful, efficient and creative labor (4). Time management ability refers to such an ability to manage time that people have, and the key to time management lies in people’s management of themselves (5), minimizing the waste of time and maximizing the utility of time (6). Efficient time management has become the foundation of nursing, and the key to cultivating talents to meet the needs of the nursing profession lies in improving the quality of nursing education (7), so we need to focus on the cultivation of nursing staff’s time management ability.

In 1989, in order to measure time management skills, Britton (8) and others proposed a theoretical model of time management from the perspective of information processing, and Britton later developed a time management questionnaire based on this model (9). Macan et al. (10) developed the Time Management Behavior Scale consisting of four dimensions: setting goals and priorities, implementing time management mechanisms, establishing organizational bias, and controlling time perception in 1990. Some studies have shown (11) that clinical nurses have good time management skills at work, which can reduce their work stress and lead to better physical and mental health.

In China, Huang et al. (12) developed the Adolescent Time Management Tendency Scale applicable to adolescents. Subsequently, in 2015, Yuan (13) revised the scale and adapted it to a time management scale applicable to employee groups. However, due to the specificity of nursing staff’s work, no time management scale developed for hospital workers has been found in China. Zyoud (14) 2023 developed a time management scale for nursing staff that can accurately and effectively measure the level of time management of nurses at work. Therefore, this study introduced the Nurses’ Time Management Scale into China and tested its reliability and validity, and then investigated the current level of time management of nursing staff in China, and proposed opinions and measures to improve their work efficiency.

## 2 Materials and methods

### 2.1 Participants

Convenience sampling method was used to select clinical nurses for the survey from June to September 2023 in two hospitals in Shandong Province. Inclusion criteria: (1) Chinese registered nurses; (2) engaged in clinical nursing; (3) informed consent and voluntary enrollment in the study. Exclusion criteria: (1) nurses in training and internship; (2) Practicing or further training nurses; (3) Dropouts. The sample size for exploratory factor analysis is at least 5–10 times the number of scale items, and the sample size for validation factor analysis is not less than 200 (15). In this study, the sample size was appropriately enlarged in consideration of 10% invalid questionnaires, so that 350 patients were finally surveyed and 345 were validly recalled, with a valid recall rate of 98.57%. At the time of the survey, three uniformly trained researchers retained the contact numbers of those who volunteered to participate in the retest to fill out the survey, which was repeated 2 weeks later (52 questionnaires were distributed and 50 were validly returned, with a valid return rate of 96.2%) in order to calculate the retest reliability coefficient.

### 2.2 Instruments

#### 2.2.1 The general information questionnaire

The patients’ gender, age, education, years of work, marital status, and title were collected.

#### 2.2.2 Nursing Time Management Scale

The scale was developed by Zyoud (14) to be able to reflect the time management skills of nurses in their clinical work. It consists of three dimensions with a total of 17 entries, namely planning activities and setting goals (entries 1–6), coordinating activities and procedures (entries 7–10) and organizing nursing activities (entries 11–17). The scale is based on a 5-point Likert scale, and the total score of the scale ranged from 17 to 85, with higher scores indicating that nurses were better at managing their time. The Cronbach′s alpha coefficient for the original scale was 0.953.

### 2.3 Procedures

#### 2.3.1 Forward and back translation

The researchers contacted the authors of the original scale and obtained their consent to translate and use the scale. The nurses’ time management competence scale was translated and back-translated in strict accordance with the Brislin translation-back translation model (16). This study followed the guidelines for translation and cross-cultural adaptation of nursing research in China (17). (1) Direct translation: A nursing postgraduate student with study abroad experience and a master’s degree in English were invited to translate independently to obtain the first drafts A1 and A2. (2) Integration: The Chinese version of the Nurses’ Time Management Scale (NTMS) A1 and A2 were compared and integrated with the original scale by members of the group, and the Chinese version of the NTMS 1.0 was developed through discussion and negotiation with the translators and the researcher. (3) Back-translation: A doctoral student in nursing with clinical experience and a doctoral student majoring in English independently translated NTMS 1.0 into English B1 and B2 without having seen the original English version of the Nurses’ Time Management Scale. (4) Re-integration: Members of the group compared and integrated the English versions of B1 and B2 with the original scale, and formed the English version of the NTMS 2.0 after discussion and negotiation with the research team. (5) Review by the original authors: The English version of NTMS2.0 was sent to the authors of the original scale, and the entries were semantically consistent with the original scale and recognized by the original authors.

#### 2.3.2 Cultural adaptation

In accordance with the cross-cultural (18) debugging process of the scale, seven experts in the related fields of clinical medical-surgical nursing and psychology were invited to evaluate the entries in terms of their contextual background. They evaluated the entries in terms of the contextual background of the entries, the language expression habits, and the applicability of the entries. For the disagreeable entries, the research team made modifications, The research team replaced “Administration” with “management,” taking into account the fact that the original scale used “Administration,” which, in the opinion of the experts, has an ambiguous semantic meaning. Until all the entries reached consensus, forming NTMS 3.0.

#### 2.3.3 Pre-experiments

The researchers selected 50 clinical nurses who met the inclusion criteria for the pre-survey and were consulted on changes to the scale as a whole, the content of the entries, and the scoring methodology, and amendments were made to unclear or controversial statements.

### 2.4 Data collection

Prior to the study investigation, consent and co-operation was obtained from the nursing department of the hospital. The research team recruited study participants from different departments in different hospitals and used a uniform guideline to explain the purpose of the study and inform the precautions for completion. QR codes and links were distributed through Questionnaire Star, which were filled out online by the respondents.

### 2.5 Statistical analyses

SPSS 25.0 and AMOS 24.0 software were used for data entry and analysis. Measurement data were described by mean ± standard deviation, and count data were described by frequency and percentage. Item analyses included critical ratio method and correlation coefficient method. Validity tests used content validity and structural validity. Among them, content validity used the content validity index at scale level and item level; structural validity used exploratory factor analysis (EFA) and confirmatory factor analysis (CFA). Reliability tests included Cronbach’s alpha coefficient, folding reliability, and retest reliability. The results were considered statistically significant at *p* < 0.005.

## 3 Results

### 3.1 Research subject characteristics

Of the 345 study participants, consisted of 45 (13.0%) were male, 300 (87.0%) were female, 123 (35.7%) were aged <30 years, 131 (38.0%) were aged 30–40 years, 91 (26.4%) were ≥ 40 years. Other general information is shown in Table 1.

**Table 1:** Frequency distribution of demographic characteristics (*n* = 345).

Characteristics	*n*	%	t/F	*p*
Gender				
Male	45	13.0	0.418	0.676
Female	300	87.0		
Age (years)				
<30	123	35.7	2.053	<0.001
30–40	131	38.0		
≥40	91	26.4		
Education				
Specialized and below	66	19.1	1.265	0.117
College	181	52.5		
Master and above	98	28.4		
Working years				
<5	107	31.0	1.285	0.102
5 ~ 10	148	42.9		
≥10	90	26.1		
Marital Status				
Married	169	56.8	1.612	0.008
Unmarried	122	35.4		
Other	27	7.8		
Title				
Nurse	85	24.6	2.315	<0.001
Nurse practitioner and above	260	75.4		

### 3.2 Item analysis

#### 3.2.1 Critical ratio method (critical ratio, CR)

Clinical nurses’ total scores on the scale were ranked from high to low, with the top 27% defined as the high subgroup and the bottom 27% as the low subgroup, and independent samples t-tests were used to determine the differences in scores between the high and low subgroups on the 17 items, with the respective critical ratios ranging from 9.365 to 21.364, and with a statistically significant difference (*p* < 0.001).

#### 3.2.2 The question total correlation method

Correlation coefficients between individual entries and the total score were calculated, and when *r* > 0.4, the entries were retained as indicating high homogeneity with the overall scale. The correlation coefficients between the entries and the total score of the scale in this study ranged from 0.127 to 0.981 (*p* < 0.01).

### 3.3 Construct validity

#### 3.3.1 Exploratory factor analysis

**Figure 1:**
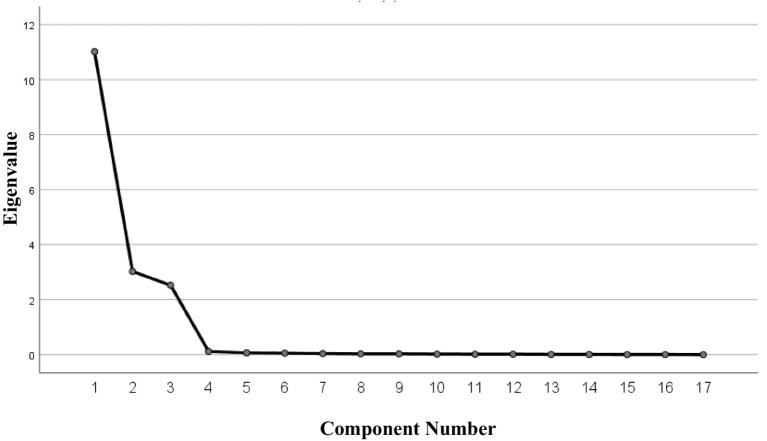
Screen plot of exploratory factor analysis for the Chinese version of the NTMS scale (*n* = 133).

The tested KMO value was 0.84 and the Bartlett’s sphere test approximate chi-square value was 6394.47 (*p* < 0.001), which was suitable for factor analysis (19). Without the requirement of the number of factors, this study extracted a total of three factors with each eigenroot greater than 1. Combined with an abrupt steepening of the gravel plot slope line into three factors (Figure 1), the cumulative variance contribution rate was 97.44% (Table 2).

**Table 2:** Factor loadings of exploratory factor analysis for the Chinese version of the NTMS scale (*n* = 133).

Items	Factor 1	Factor 2	Factor 3
Q1. I assign myself a set of goals each day.		0.935	
Q2. I have complete goals for the week.		0.939	
Q3. I force myself to make time to plan.		0.939	
Q4. I have enough time to plan.		0.937	
Q5. I have time to think about how to turn my plans into action.		0.935	
Q6. I know exactly what I want to accomplish during the day and make a list of activities.		0.932	
Q7. I coordinate for medication management.			0.940
Q8. I coordinate the administration of treatments.			0.946
Q9. I coordinate for nursing procedures.			0.939
Q10. I am able to develop shift handover reports.			0.937
Q11. I keep the work area tidy and keep it neat and tidy.	0.927		
Q12. I group activities in the same place.	0.920		
Q13. I prepare all equipment needed before starting an activity.	0.931		
Q14. I estimate the time needed to complete the task.	0.941		
Q15. I record nursing actions in a timely manner after the activity is completed.	0.938		
Q16. I process nursing paperwork efficiently.	0.940		
Q17. I use appropriate technology to facilitate communication and coordination.	0.942		
Eigenvalue	11.02	3.03	2.52
Explained variance (%)	39.347	34.531	23.566
Cumulative variance contribution (%)	39.347	73.878	97.444

#### 3.3.2 Confirmatory factor analysis

**Figure 2:**
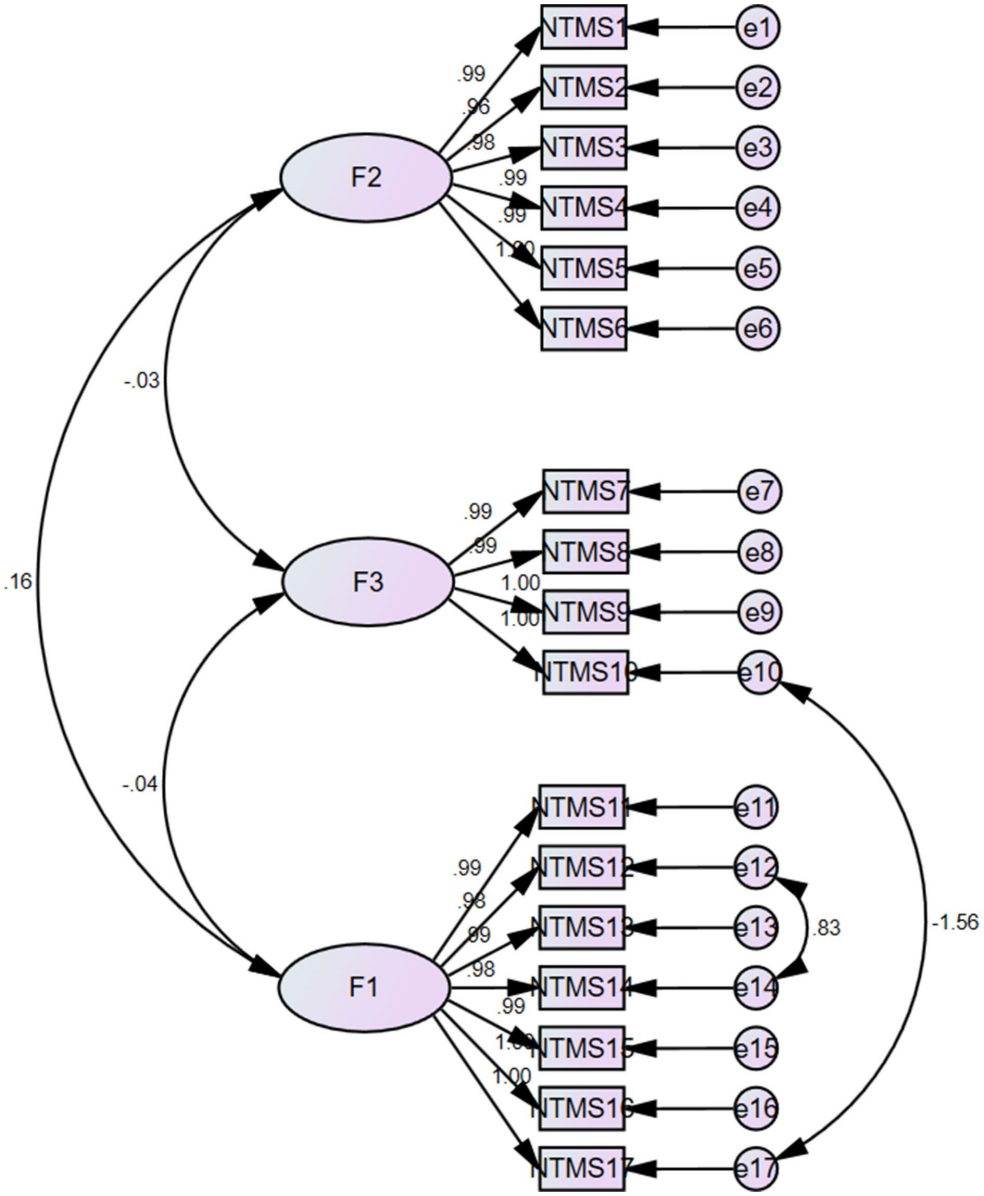
Standardized three-factor structural model of the NTMS scale (*n* = 212).

The three dimensions delineated by the exploratory factor analysis were further validated to obtain the initial model. The results of the analysis showed X^2^/df = 2.610, RMSEA = 0.019, NFI = 0.975 and CFI = 0.985 (Table 3). The structural equation model was shown in Figure 2.

**Table 3:** The model fitness index of confirmatory factor analysis of the Chinese version NTMS scale (*n* = 212).

Item	X^2^	df	X^2^/df	RMSEA	NFI	CFI	TLI	IFI
Criteria	—	—	1 ~ 3	<0.1	>0.9	>0.9	>0.9	>0.9
Indices	297.508	114	2.610	0.019	0.975	0.985	0.982	0.985

### 3.4 Content validity

The validity of the scale content was assessed using an expert rating method. Seven experts were invited to evaluate the Chinese version of the Nurses’ Time Management Competency Scale on a 4-point Likert scale. The content validity indices of the scale items were calculated based on the results of the experts’ deliberations: I-CVI of 0.86 to 1.00, S-CVI of 0.94, and S-CVI/Ave of 0.99, indicating good content validity of the scale (20).

### 3.5 Reliability validity

The Cronbach’s alpha coefficient for the Chinese version of the NTMS scale was 0.966; the retest reliability was 0.951, indicating that the scale had good reliability (Table 4).

**Table 4:** Chinese version of the NTMS scale reliability results.

Variables	Cronbach’s alpha coefficient	Split-half reliability	Test–retest reliability
NTMS scale	0.966	0.771	0.951

## 4 Discussion

In the Chinese version of the scale, in order to adapt to the Chinese national conditions and cultural background, the researchers had repeated deliberations and considerations when localizing some of the indicators of the scale. For example, when some indicators differed from the current work behaviors of Chinese nursing staff, they were changed appropriately after expert consultation, and the Chinese version of the Nurses’ Time Management Scale was finally formed. In this study, the Arabic version of the Nurses’ Time Management Scale was Chineseized into the Chinese version of the Nurses’ Time Management Scale, and a study was conducted on some nurses in northern China to investigate the time management level of Chinese nurses. The scale consists of three dimensions, namely organization, coordination and planning, with a total of 17 entries, which is similar to the structure of the original scale with good reliability and validity.

Some studies have shown (21), hospital nurse managers have good time management ability, efficient time management can improve the performance management of nurse managers, nurse managers have clear work objectives and prioritized level classification according to the work objectives. The work of nursing management mainly focuses on the two aspects of daily management and human resource management (6), and nurses’ clinical work is affected by the management of daily affairs and human resource management. Improving the time allocation and time priorities of nursing staff can promote the quality and effectiveness of patient care. The acquisition of time management skills by new nurses is a key component in reducing turnover rates and improving work well-being (1). Heavy clinical workloads may prevent nursing from becoming a more specialized discipline (22), and nurses should ensure that they have sufficient time to focus on nursing supervision and improve their time utilization. Research has shown that a manager’s time management can affect not only productivity and organizational success, but also an individual’s life-work balance (23). Time management skills can help nurse leaders meet challenges and improve the quality of care (24).

The time management level of Chinese nursing staff is at a medium-high level. Zyoud (5) conducted a survey on healthcare workers in the northern part of Palestine, and the respondents’ time management skills were relatively good, with different genders, different hospital grades, different titles, and whether or not they had attended a time management course as factors affecting nurses’ time management skills. The results indicated that Chinese nursing staff were able to rationalize their schedule for the day based on their daily workload. In order to promote patient care satisfaction and patient safety, it is recommended that nurses with low levels of time management should take further education or integrated care management (25) to form an interdisciplinary team, which will ultimately lead to efficient and precise quality of care.

In this study, the critical ratio of the questionnaire entries was 9.365 ~ 21.364, which was higher than the standard value, suggesting that the scale had a better discriminatory ability; the scores of each entry were moderately to highly correlated with the total score of the scale, which indicated that the scale was able to assess the degree of protection of the patient’s privacy better, and the representativeness was good. In addition, the Cronbach′s α coefficient after deletion of each entry did not exceed the initial value of the translated scale. The results of item analyses indicated that the Chinese version of the NTMS had a high degree of discrimination and representativeness and did not require the deletion of any entries.

Reliability can reflect the reliability and stability of a scale (26). In this study, the reliability of the Chinese version of the NTMS was analyzed in terms of three dimensions: Cronbach′s α coefficient, folded reliability and retest reliability. The results showed that the Cronbach′s α coefficient of the Chinese version of the NTMS was 0.966, which was higher than the results of the Arabic version (14), and the Cronbach′s α coefficients of the dimensions ranged from 0.920 to 0.946. Folded-half and re-test reliabilities were 0.771 and 0.951, respectively, indicating that the reliability of the scale was good (27), and the stability was high. The Chinese version of the NTMS can be used for the measurement of nurses’ time management level, guiding nursing managers to develop a more perfect management system and improve the quality of nursing work.

Content validity is often used to test whether the designed items are representative of the content or topic to be measured and to judge whether the content of the scale meets the objectives of the study (20). The I-CVI of the Chinese version of the PPS was 0.86–1.00, the S-CVI was 0.94, and the S-CVI/Ave was 0.99, which showed good content validity (28), suggesting that the entries could accurately measure nurses’ time management ability. The content of the Chinese version of the NTMS can accurately reflect the time management level of nursing staff at work, improve work management efficiency, and appropriately arrange work behaviors throughout the day, so that patients can receive more comprehensive and efficient nursing services.

Exploratory factor analysis is used to explore the dimensions and structures involved in each entry of the scale (29), and a cumulative variance contribution of >40% for the common factors and a corresponding factor loading value of >0.4 for each entry indicate that the scale has good structural validity. In this study, exploratory factor analysis extracted three factors that explained 97.44% of the total variance, and the attribution of the entries remained consistent with the original scale. Validatory factor analysis was used to further validate the model, and the results showed that the fitting indexes of the Chinese version of the NTMS were in accordance with the regulations (30). In summary, the Chinese version of the NTMS has ideal validity in the Chinese nurse population.

## 5 Limitations

The study has some limitations. First, this study only recruited clinical nurses from a northern province, and no study of this size has been conducted in the south, which may affect its representativeness. Second, only a cross-sectional survey was conducted in this study, and a multicenter, large sample study on a larger scale is needed.

## 6 Conclusion

In this study, rigorous translation criteria and cultural adaptations of the NTMS scale were performed to produce a Chinese version of the NTMS scale, which has good reliability and measures the time management skills of Chinese clinical nurses in clinical work. This study provides a method to further improve nurses’ management skills, aiming to improve nurses’ time allocation skills in clinical work. The Chinese version of the Nurses’ Time Management Scale can enable nursing staff to rationally allocate their working time, improve work efficiency, and enable patients to receive perfect health care services.

## Data availability statement

The raw data supporting the conclusions of this article will be made available by the authors, without undue reservation.

## Ethics statement

The studies involving humans were approved by the Ethics Committee of Jinzhou Medical University. The studies were conducted in accordance with the local legislation and institutional requirements. The participants provided their written informed consent to participate in this study.

## Author contributions

ZF: Data curation, Investigation, Supervision, Validation, Writing – original draft, Conceptualization. YW: Data curation, Investigation, Methodology, Resources, Visualization, Writing – original draft. LZ: Data curation, Investigation, Writing – original draft. MT: Data curation, Investigation, Software, Writing – review & editing.
